# Crystalline Modification and Its Effects on Dielectric Breakdown Strength and Space Charge Behavior in Isotactic Polypropylene

**DOI:** 10.3390/polym10040406

**Published:** 2018-04-05

**Authors:** Ling Zhang, Yunxiao Zhang, Yuanxiang Zhou, Chenyuan Teng, Zhaowei Peng, Stephen Spinella

**Affiliations:** 1 State Key Laboratory of Control and Simulation of Power Systems and Generation Equipments, Department of Electrical Engineering, Tsinghua University, Beijing 100084, China; zhangling15@tsinghua.edu.cn (L.Z.); zhangyxthu@gmail.com (Y.Z.); pzw16@mails.tsinghua.edu.cn (Z.P.); 2 State Key Laboratory of Electrical Insulation and Power Equipment, Xi’an Jiaotong University, Xi’an 710049, China; 3 School of Electrical Engineering, Xinjiang University, Urumqi 830047, China; 4 School of Electrical Engineering, Wuhan University, Wuhan 430072, China; tengchenyuan@126.com; 5 Department of Chemical and Biomolecular Engineering, NYU Polytechnic School of Engineering, 6 MetroTech Center, Brooklyn, NY 11201, USA; sgaluf@gmail.com

**Keywords:** isotactic polypropylene, nucleating agent, crystalline modification, impact strength, dielectric breakdown strength, space charge

## Abstract

Adding nucleating agents (NAs) is one of the most efficient ways to obtain improved mechanical, optical, and thermal properties of isotactic polypropylene (iPP). While it is well appreciated that electrical property is critically affected by crystalline modification, the role between them remains unclear. Here, we address this issue by incorporating commercial α-NA and β-NA into iPP, both of which exhibit strong nucleation ability, e.g., reducing the size of crystalline agglomerates from 45.3 μm (Pure-iPP) to 2.5 μm (α-iPP) and 7.6 μm (β-iPP), respectively. Mechanical testing results show that while β-modification decreases the tensile strength a little, it does enhance the elongation at break (200%) and toughness (25.3% higher), relative to its unfilled counterparts. Moreover, a well-dispersed β-iPP system obtains a comprehensive improvement of electrical properties, including dielectric breakdown strength, space charge suppression, and internal field distortion under a high external field (−100 kV/mm) due to newly-generated deep charge trapping sites. This crystalline modification strategy is attractive for future development of many engineering insulating polymers.

## 1. Introduction

Isotactic polypropylene (iPP) exhibits a combination of good comprehensive properties, i.e., excellent insulation performance, stable thermal property, high chemical durability, easy processability, and low price, and is therefore one of the most widely used thermoplastic polymer [1]. iPP can crystallize in three major crystal forms, including monoclinic α-crystal [2], trigonal β-crystal [3], and orthorhombic γ-crystal [4]. Thermodynamically stable α-crystal is mainly obtained under standard processing conditions [5]. α-Nucleating agents (α-NAs) can improve the stiffness and transparency properties of iPP materials but decrease its ductility. In comparison, β-crystal is thermodynamically less stable than α-crystal, but can be induced by shear [6,7], temperature gradient [8,9], or adding special β-NAs [10,11]. Adding β-NAs is the most common and effective way to achieve a high relative content of β-crystal in iPP [12]. β-crystal endows iPP products with better toughness properties, which shows great prospects in industrial production [13]. For example, iPP has been regarded as a potential insulating material candidate to replace crosslinked polyethylene in the field of high voltage (HV) extruded cable insulation in recent years, for it is non-crosslinking, recoverable, and environmentally friendly [14,15,16]. However, the application is still limited, due to its low impact strength, especially under low temperature conditions. 

Currently, tremendous research has been carried out to improve the toughness of iPP to broaden its industrial application and adding fillers, i.e., flexible polymers and NAs, is the easiest and most industrially relevant method to toughen iPP [17,18,19,20], but electrical property and toughness performance are usually compromised. β-modification is often adopted during processing to accelerate the nucleation process and to control the crystal morphology of iPP [21]. The toughening effect of β-NA has been explained by different morphologies of the spherulites, and different models have been proposed to explain the higher toughness of β-iPP. To name a few, Riekel reported that β-modification is accompanied by the appearance of microvoids, resulting in higher toughness of the material [22]. Koteck suggested a model based on a higher continuity of the amorphous phase in the presence of crystal lamellae with β-modification, resulting in higher toughness of β-iPP [23]. Aboulfaraj investigated the brittle performance of α-iPP compared to β-iPP by the different ability of crystallites under mechanical loadings due to the different structures of the spherulites [24].

As for the effect of crystalline modification on electrical property, Zheng reported that the fast cooling process contributed to the generation of shallow trap level and higher mobility of the escaping charge in iPP films [25]. Dang simultaneously added elastomer and surface-modified ZnO nanoparticles into PP and obtained improved DC resistivity and space charge properties [26]. Wu reported the suppression of space charge in iPP by inducing the β-crystal formation [27]. Zha found that γ-crystal could induce deep traps, resulting in the decrease of charge accumulation and electrical conductivity of copolymerized ethylene–propylene composites [28]. While this knowledge exists, comprehensive investigation has not yet been carried out to quantitatively study the effect of crystalline modification, i.e., α-NAs and β-NAs, on morphology behavior, mechanical property, dielectric breakdown strength, and internal field distortion under high external fields in iPP.

For this purpose, our main objective is to investigate the enhancement mechanism of electrical property and space charge characteristics of β-iPP. Space charge measurements were carried out at room temperature under −100 kV/mm based on the pulsed electroacoustic (PEA) method. For comparison purpose, pure iPP and α-iPP (containing 0.2 wt % commercial α-NA) were fabricated as reference groups. The microstructure and crystal morphology were investigated. The influence of α-NA and β-NA on the thermal and electrical properties, especially space charge evolution of iPP, was synchronously investigated. Furthermore, the compromised relationship between tensile property and impact strength of β-iPP was discussed.

## 2. Materials and Methods

### 2.1. Materials

iPP pellets (CAS 9003-07-0) were supplied by Xiya Reagent Company, China, with the melt flow index of 4 g/10 min (230 °C/2.16 kg), the melting point temperature of 160–165 °C, and the density of 0.9 g/mL at 25 °C (lit.). Aryl amide derivative TMB-5 β-NA, existing in the form of white powder, was kindly provided by Shanxi Chemical Research Institute (Co., Ltd., Taiyuan, China). From the data sheet, TMB-5 β-NA is suitable to simultaneously improve the impact strength and heat distortion temperature with the recommended mass fraction of 0.1–0.3 wt %. TMP-6 α-NA, in the form of white powder, was also from Shanxi Chemical Research Institute (Co., Ltd.), with the main composition of substituted aromatic phosphates. The highlighted feature of TMP-6 α-NA are able to largely enhance the rigidness and transparency of iPP materials with the recommended mass fraction of 0.1–0.3 wt %.

### 2.2. Composites and Sample Preparation

Before mixing, all materials were vacuumed at 80 °C for 48 h, to lower water content as much as possible. iPP pellets and 0.2 wt % TMB-5 β-NA were physically premixed at 500 rpm for 5 min via a motor stirrer, in order to fabricate β-iPP samples. Then, melt blend mixing was conducted via a SJSZ-10A twin screw extruder (the L/D ratio, the length, and the diameter (big end) of the screw are 7.9, 190 mm, and 24 mm, respectively; Wuhan Ruiming Plastic Machinery Co., Ltd., Wuhan, China) at 180 °C and 36 rpm for 20 min under N_2_ atmosphere. Compression molding was carried out in a press (QLB-100T, Taizhou Xiangxing Rubber and Plastic Machinery Co., Ltd., Wuxi, China) at 180 °C under 10 MPa for 10 min with polyimide substrates, and subsequently, film samples were annealed via air cooling naturally. Then, film samples were vacuumed and shorted at 80 °C for another 48 h to eliminate water content, residual stress, local orientation, and the charge generated during the compression molding process. For comparison purpose, α-iPP and pure iPP (denoted as Pure-iPP) were fabricated in the same procedure. The dimension of samples is 120 mm × 90 mm × 200 μm.

### 2.3. Methods

#### 2.3.1. Field Emission Scanning Electron Microscopy-EDX

Field emission scanning electron microscopy (FESEM) was performed using a JSM-6335 (JEOL Ltd., Tokyo, Japan) coupled with an energy dispersive X-ray (EDX) to observe the morphology of NAs and fractured faces of iPP samples, and to determine the elemental composition of both the surface and bulk features. Brittle fracture sections of iPP samples were obtained in liquid nitrogen and coated with platinum with a sputter time of 6 min and 25 mA current, before being inserted into the specimen chamber. During the FESEM observation and EDX analysis, the voltage was set as 15 kV.

#### 2.3.2. Polarizing Optical Microscopy (POM)

iPP films (~20 μm thick) were specially prepared between glass slides at 200 °C for POM observation to distinguish the crystalline structure of α-crystal and β-crystal morphology of iPP samples. The POM equipment was purchased from Shanghai Optical Instrument Factory No. 1. Ltd., Shanghai, China. Observation mode and the magnification were set as polarization mode and ×500, respectively. Specially, there is no polarizer used during the testing.

#### 2.3.3. X-ray Diffraction (XRD)

A Rigaku SmartLab XRD system was adopted to study the crystalline structure of iPP samples. The wavelength is set as 1.5418 Å, the voltage as 40 kV, and the current as 40 mA. XRD pattern was recorded over angles ranging from 10° to 35° with a scan rate of 4 °/min. The relative content of β-crystal, *K*_β_, could be calculated using Tuner–Jones formula [29].

(1) $$K_{\beta }=\frac{A_{\beta (300)}}{A_{\beta (300)}+A_{\alpha (110)}+A_{\alpha (040)}+A_{\alpha (130)}},$$

where $A_{\beta (300)}$ represents the diffraction intensity of β(300) plane at diffraction angle 2θ = 16.1°. $A_{\alpha (110)}$, $A_{\alpha (040)}$, and $A_{\alpha (130)}$ represent the diffraction intensities of α(110), α(040), and α(130) planes at diffraction angles 2θ = 14.1°, 16.9°, and 18.5° in the XRD pattern, respectively.

#### 2.3.4. Differential Scanning Calorimetry (DSC)

The effect of NAs on the thermal property of iPP was investigated using Q100 DSC measurement manufactured by TA Instrument Ltd., New Castle, DE, USA. The mass of the samples were in the range of 5–7 mg. The heating and cooling process is performed under a N_2_ flow. Melting behavior can reflect the crystalline structure, which is very important in analyzing the crystal distribution. The temperature range was set as 20–200 °C, and both the heating and cooling rates were 10 °C/min. The samples were heated and cooled twice, and only the second round of heating and crystallization curve was selected, in order to avoid previous thermal history. Temperature readings and calorific measurements were calibrated via an indium standard.

The percentage of β-crystal of iPP samples, $\Phi_{\beta }$, could be calculated according to [30]:

(2) $$\Phi_{\beta }=\frac{\chi_{\beta }}{\chi_{\beta }+\chi_{\alpha }},$$

where $\chi_{\alpha }$ and $\chi_{\beta }$ are the crystallinities of α-iPP and β-iPP, which were determined according to

(3) $$\chi_{i}=\frac{\Delta H_{i}}{\Delta H_{i}{}^{\theta }}\times 100\%,$$

where ∆*H_i_* is the calibrated specific fusion heat of α-crystal or β-crystal. $\Delta H_{i}{}^{\theta }$ is the standard fusion heat of α-iPP (178 J/g) or β-iPP (170 J/g) [31,32].

The specific fusion heats for α-crystal and β-crystal were determined according to an approximate method proposed in literature, due to the simultaneous existence of both α and β fusion peaks in some samples [33]. Due to partial β-crystal transformation into α-crystal during the DSC scan, leading to the increase of $\Phi_{\alpha }$, the relative content of β-crystal obtained by DSC measurement will be lower than the value measured by XRD [34]. However, the variation trends of both α-iPP and β-iPP can be achieved, and the data are acceptable to some extent.

#### 2.3.5. Mechanical Property Measurement

Tensile, flexural, and Izod impact properties of iPP samples were tested at room temperature (around 25 °C) according to ASTM D638, ASTM D790, and ASTM D256, respectively. The model of tensile and flexural tester and Izod impact tester was WDW-1 and JB-50B respectively, both of which were manufactured by Jinan Shijin Group Co., Ltd., Jinan, China. The reported mechanical strength is the average of at least three specimens of each group.

The dimension of dumbbell-shape specimens for tensile tests is 165 mm × 19 mm × 2 mm, with a middle-width of 13 mm. The dimension of cuboid specimens for flexural tests is 50 mm × 12 mm × 2 mm, and the dimension of specimens for Izod impact tests is 120 mm × 10 mm × 10 mm. For tensile tests, the test rate is 50 mm/min. The distance between supports for the flexural properties is 25.4 mm. The energy of hammer for the Izod impact test is 50 J. 

#### 2.3.6. Electrical Property Measurement

DC conduction of iPP samples has been measured with a lab-made three-electrode unit and Kiethley6517A picoammeter at room temperature according to IEC standard 60093. Film samples (~200 μm thick) were coated with gold electrodes using EDS3000 coater (Beijing Elaborate Technology Development Ltd., Beijing, China). DC field was selected as 20 kV/mm and poling time was 300 s, which was regarded long enough to reach quasi-steady state.

Film samples (~100 μm thick) were used for negative and positive DC breakdown strength (BDS) tests, which were carried out with lab-made ball-plane electrodes at room temperature with a ramp rate of 500 V/s, according to International Electrotechnical Commission (IEC, Geneva, Switzerland) standard 60243-2: 2001. Two-parameter Weibull distribution is adopted to evaluate DC BDS.

Space charge measurement was performed based on the pulsed electroacoustic (PEA) method [35]. A lab-made solid-state high voltage (HV) pulse generator was applied, with the main parameters of 400 Hz, 400 V, and 5 ns, referring to repetition frequency, pulse magnitude, and pulse width, respectively. The polarization process was carried out under −100 kV/mm for 30 min, and the depolarization process for 5 min with the measurement interval of 3 s.

## 3. Results and Discussion

### 3.1. Crystal Structure Characterization

**Figure 1:**
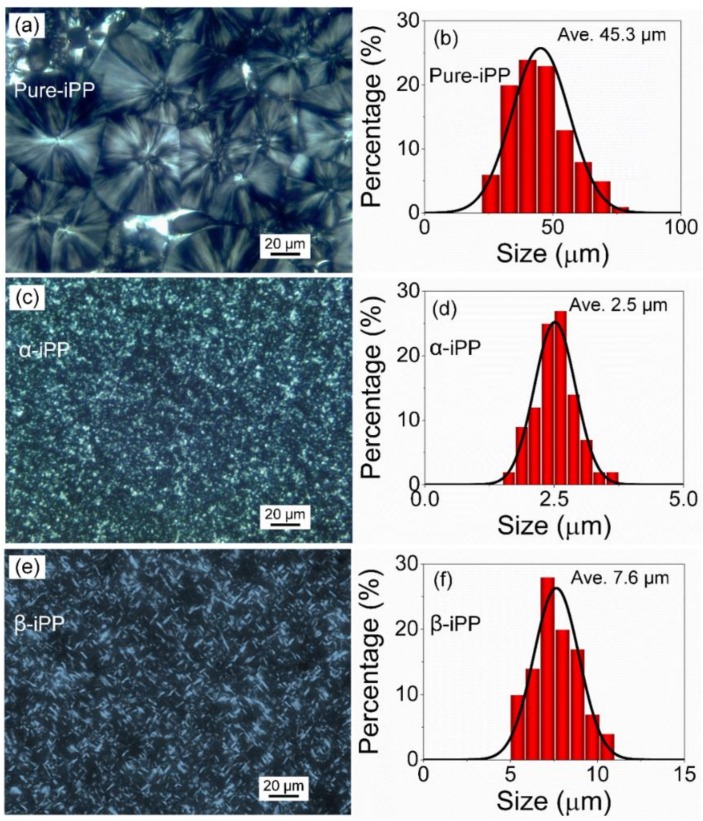
POM images and the size distribution of crystalline agglomerates of (**a**,**b**) Pure-iPP, (**c**,**d**) α-iPP, and (**e**,**f**) β-iPP.

In order to investigate the nucleation ability of β-NA and α-NA on iPP, POM observation was utilized, and the results are shown in Figure 1. In Figure 1a, only α-spherulite exists in Pure-iPP with the average size of crystalline agglomerates of 45.3 μm. It is also clearly shown that each α-spherulite has a randomly distributed nucleating center, and forms a competitive relation attracting iPP polymer chains during the growth of α-spherulite in the crystallization process. As for α-iPP and β-iPP, shown in Figure 1b,c, more nucleation sites were formed, resulting in much smaller and narrower size of crystalline agglomerates, i.e., 2.5 μm for α-iPP and 7.6 μm for β-iPP, indicating that TMP-6 and TMB-5 indeed acted as effective NAs in the iPP matrix. Moreover, the morphologies of α-crystal and β-crystal were quite different due to the typical crystalline mechanism of α-NA and β-NA. From POM observation, α-NA and β-NA were well dispersed in the bulk, exhibiting good compatibility with the iPP matrix, which was in agreement with FESEM results.

**Figure 2:**
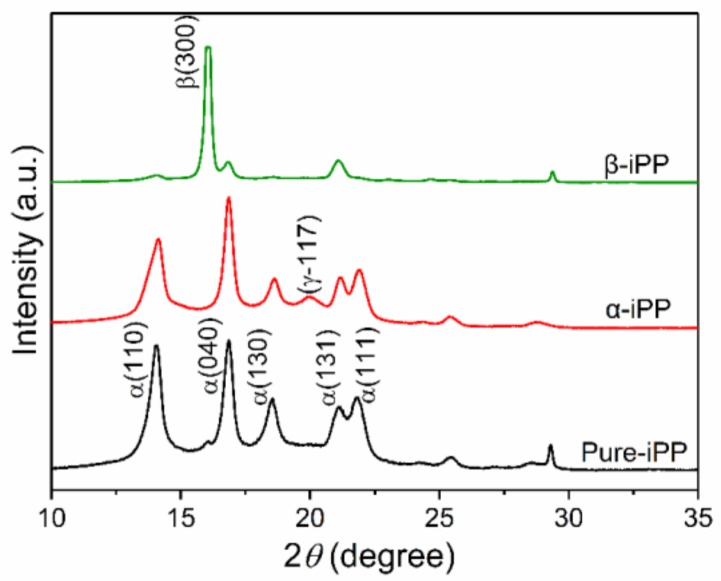
XRD patterns of iPP samples.

Figure 2 shows the XRD pattern of iPP samples. The corresponding relative content of β-crystal, $K_{\beta }$, and $\Phi_{\beta }$ calculated from DSC data, is shown in Table 1. The main lattice planes corresponding to the reflection peaks are α(110), β(300), α(040), α(130), α(111), and α(131) at diffraction angle *2*θ of 14.1°, 16.1°, 16.9°, 18.5°, 21.4°, and 21.8°, respectively [36]. According to Equation (1), the introduction of β-NA increases the relative content of β-crystal to 0.81 ($K_{\beta }$), due to its strong inducement capability to form β-crystal, reflecting the strong diffraction intensity peak observed in the XRD pattern of β-iPP. By contrast, a clear α-crystal was detected in Pure-iPP and α-iPP, and a weak peak of γ(117) lattice plane was observed only in α-iPP. From the “structure-property” relation perspective, it can be concluded that the macroscopic insulation performance and mechanical property are inextricably related with the crystal structure and distribution when NAs are introduced into the iPP matrix. Moreover, a comprehensive measurement of thermal property was carried out.

### 3.2. Morphology Characterization

**Figure 3:**
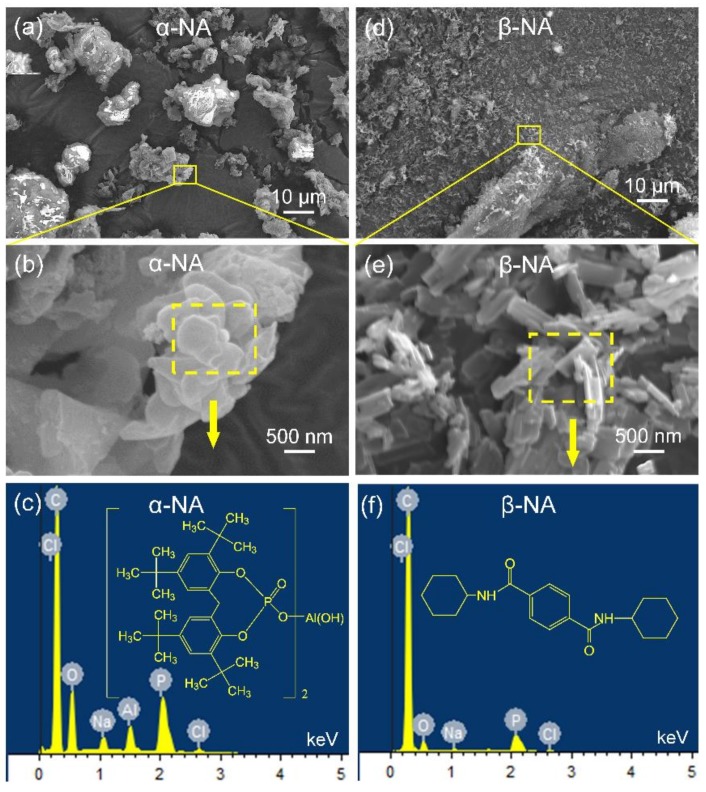
Geometric shapes and element compositions of NAs are observed by FESEM and EDX: (**a**–**c**) α-NA, (**d**–**f**) β-NA.

Geometric shapes and element compositions of NAs are observed shown in Figure 3. 

As can be seen from Figure 3, the size of these two NAs are also different from each other, and can be classified into two groups: according to the polymorphic selectivity and geometric shape, TMP-6 is a highly efficient and flaky α-NA with the size smaller than 1 μm, and TMB-5 is a club-shaped β-NA with a length of several μm and a radius less than 200 nm. Figure 3c,f present EDX element compositions and corresponding chemical structures of TMP-6 α-NA and TMB-5 β-NA, respectively. Apart from oxygen and carbon elements, phosphorus and aluminum elements occupy a large proportion in α-NA. Besides, small amounts of sodium and chlorine elements could be detected in α-NA, probably, as well, due to the existence of impurities. As for β-NA, the amounts of sodium and chlorine elements are much less compared to those of α-NA, which might benefit the macroscopic dielectric property.

**Figure 4:**
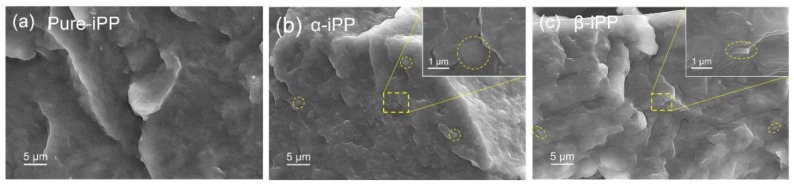
FESEM images of fractured faces of (**a**) Pure-iPP, (**b**) α-iPP, and (**c**) β-iPP. The inserted images with higher magnification represent a magnified area of the fracture surface.

Figure 4 presents SEM images of fractured faces of iPP samples. Both α-iPP and β-iPP possess a well-dispersed NA distribution in the iPP matrix (see the inset yellow dash circles in Figure 4b,c), indicating the excellent compatibility and good prospect of industrial application of these NAs. According to the POM images and previous studies, perfect α spherulites play a dominant role in the Pure-iPP samples, resulting in poor impact property for their inability to propagate the crack tip damage zone [37]. However, spherulites diminished in size and formed ordered α-crystal and β-crystal after the addition of 0.2 wt % α-NA and β-NA.

### 3.3. Thermal Characterization

**Figure 5:**
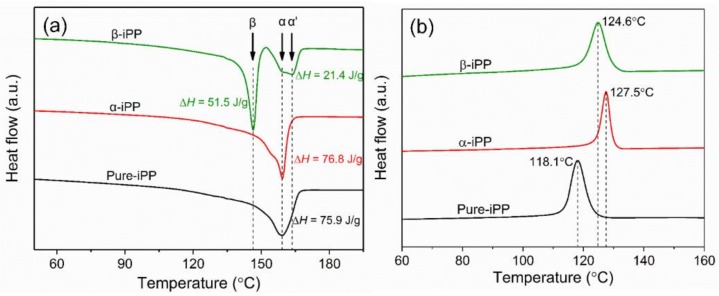
DSC results of iPP samples. (**a**) Melting curves and (**b**) crystallization curves.

Figure 5 compares the crystallization and melting behavior of iPP samples. 

Figure 5a displays the melting curves where two peaks, i.e., the low and high temperatures standing for β and α peaks, could be observed in β-iPP samples, while Pure-iPP and α-iPP only show α peaks. In addition, the melting process of iPP is closely related to their thermal stability. Figure 5a shows that α peaks of all iPP samples disappeared completely at ~168 °C, indicating that the stable α-crystal structure in iPP samples is alike. However, it is clearly shown that two similar intensity of peaks combine and form an α peak in β-iPP, indicating the presence of more stable α-crystal [38]. Figure 5b shows that both the addition of α-NA and β-NA contributes to the higher crystalline temperature from 118.1 °C (Pure-iPP) to 127.5 °C (α-iPP) and to 124.6 °C (β-iPP). From Table 1, the crystallinity of iPP samples was not affected, and stayed constant around 42.3–43.1%. Thus, an influence of the crystallinity on the electrical and mechanical properties could be excluded.

### 3.4. Mechanical Characterization

#### 3.4.1. Tensile Strength

**Figure 6:**
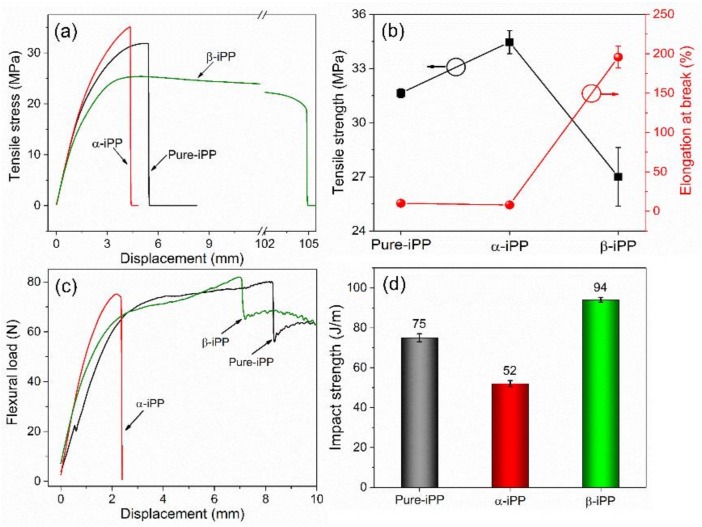
Mechanical property of iPP samples. (**a**) Relationship between tensile stress and displacement, (**b**) tensile strength and elongation at break, (**c**) relationship between flexural load and displacement, and (**d**) influence of NAs on the impact strength.

As shown in Figure 6, the tensile behavior of iPP samples with NAs are quite different from each other. The yield strength of α-iPP is higher than those of Pure-iPP and β-iPP. Meanwhile, the yielding peak width of α-iPP is obviously narrower than those of the other two groups, which might be attributed to the smaller average crystalline agglomerates size of α-spherulite. As for β-iPP, β-crystal consists of the majority of spherulites, and its yield peak width is obviously broader [39,40]. On the other hand, the elongation at break of β-iPP is about 196%, much higher than those of Pure-iPP (10%) and α-iPP (8%), indicating an excellent ductility of β-iPP, and a great prospect in the application of HV cable insulation, which might be attributed to the existence of larger proportion of β-crystal and lower amount of microvoids in β-iPP.

#### 3.4.2. Flexural Strength

For flexural strength shown in Figure 6c, similar results of β-iPP and Pure-iPP were observed, and a flexural yield load of ~82 N was obtained for β-iPP, with a relative narrower flexural yielding peak width compared to that of Pure-iPP. By contrast, α-iPP has a flexural yield load of ~75 N and was a little stiffer, resulting in the narrowest yield peak width, due to, partially, the introduction of structure defects, e.g., microvoids, in α-iPP.

#### 3.4.3. Impact Strength

Figure 6d shows the impact strength result of iPP samples. Even though the crystal morphology and average crystalline agglomerates size were completely changed, α-NA does decrease the impact strength of the iPP material from 75 J/m to 52 J/m. It can also clearly be seen that the addition of β-NA does cause a significant increase in the toughness of β-iPP samples, 20.2% higher than that of Pure-iPP. Therefore, from the viewpoint of mechanical property, it is of great significance to balance the tensile, flexural, and impact strength, e.g., adding 0.2 wt % β-NA into iPP matrix does achieve the expectant impact strength and excellent ductility, but decrease the tensile property to some extent.

### 3.5. Electrical Characterization

#### 3.5.1. DC Conduction Current

**Figure 7:**
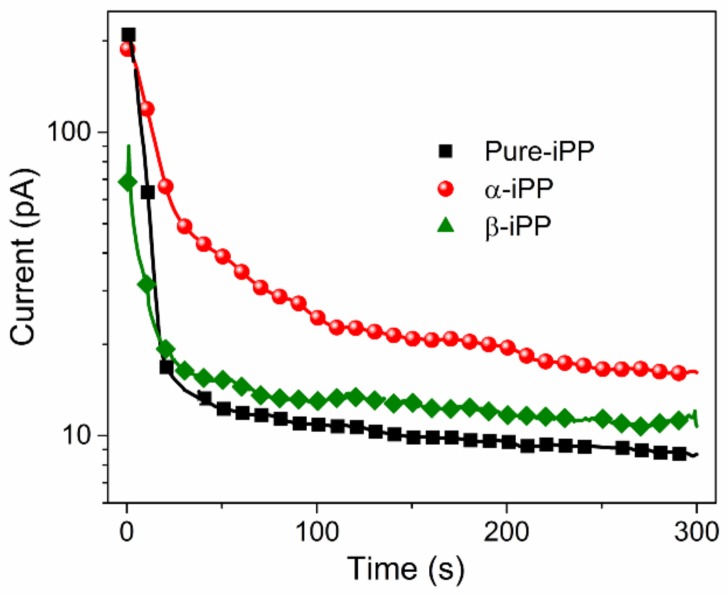
DC conduction current of iPP samples at room temperature.

Figure 7 presents DC conduction current of iPP samples under 20 kV/mm for 300 s at room temperature. Each conduction current curve consists of two parts of current components, i.e., capacitance current (mainly in the first 30 s) and leakage current (after the first 30 s). The latter is a key parameter that determines the conduction loss under DC conditions. It could also reflect the relative content of shallow trapping sites within the iPP matrix to some extent. From Figure 7, it shows that the leakage current of α-iPP is about twice of that of Pure-iPP, and β-iPP exhibits almost the same conduction current level as Pure-iPP.

#### 3.5.2. DC Breakdown Strength

**Figure 8:**
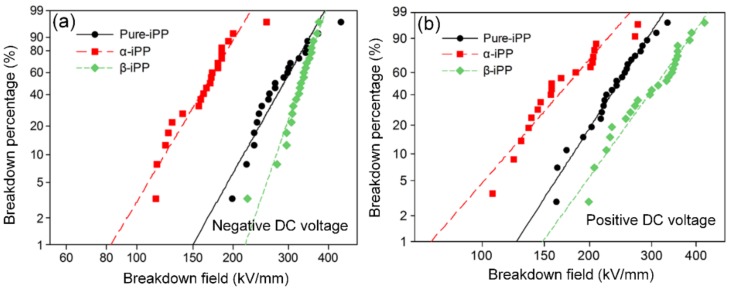
Weibull distribution of DC BDS of iPP samples. (**a**) Negative DC, (**b**) positive DC.

DC BDS tests were carried out with ball–plane electrodes and both negative and positive DC BDS were assessed by the two-parameter Weibull distribution in Figure 8 and Table 2.

The results indicate that α-iPP had the lowest DC BDS with the scale indexes of 177.4 kV/mm (negative DC) and 189.2 kV/mm (positive DC), and DC BDS of β-iPP outperformed those of Pure-iPP by 10.7% (negative DC) and 29.2% (positive DC), respectively. Besides, the shape index is a parameter which could reflect the dispersion characteristics of test results, and thus, the performance stability of material property to some extent [41]. In Table 2, both the shape indexes of negative and positive DC BDS of α-iPP are the smallest, indicating that the newly-generated microvoids probably not only lead to the decrease of DC BDS, but also the increase of instability of material performance.

#### 3.5.3. Space Charge Behavior

**Figure 9:**
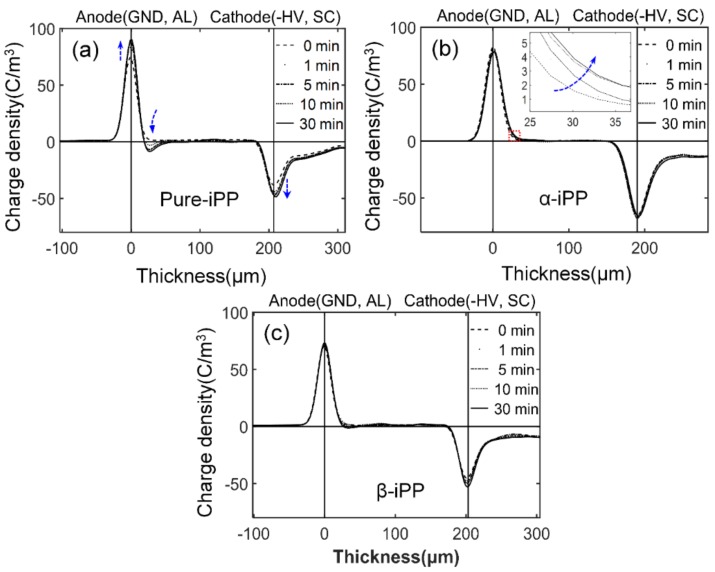
Space charge distribution in iPP samples poled under −100 kV/mm at room temperature for (**a**) Pure-iPP, (**b**) α-iPP, and (**c**) β-iPP. AL: aluminum; and SC: semiconductive layer.

Space charge distribution under −100 kV/mm was examined at room temperature shown in Figure 9. Immediately after polarization, continuously increasing net negative charges were observed in the vicinity of the anode in Pure-iPP (See Figure 9a). After being polarized for 30 min, ~10 C/m^3^ negative charge shows a concentrated distribution, and a small quantity of positive charge uniformly distributed across the bulk of Pure-iPP. By contrast, both α-iPP and β-iPP show a smaller quantity of net positive charges (less than 3 C/m^3^) and distributed uniformly within 30 min polarization.

**Figure 10:**
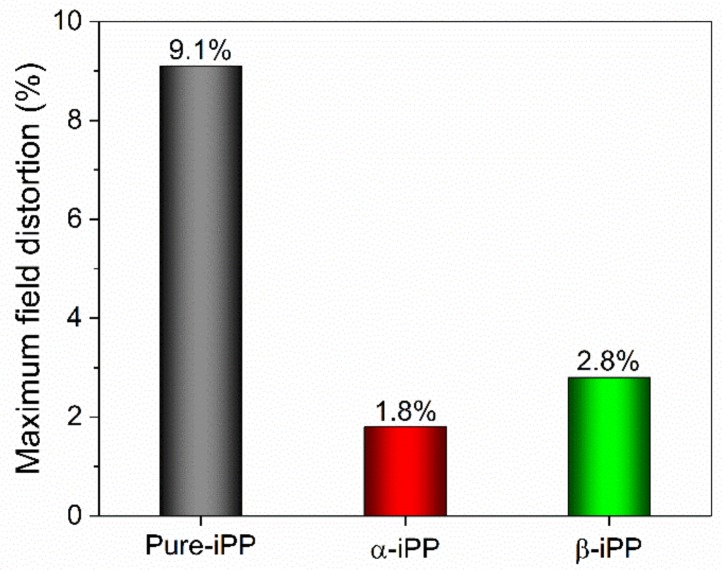
Maximum field distortion percentage in iPP samples during 60 min polarization under −100 kV/mm at room temperature.

Figure 10 presents the maximum field distortion percentage in iPP samples under −100 kV/mm within 30 min polarization, which was deduced from space charge profiles at room temperature. 

It is clearly shown that Pure-iPP encountered a severely increasing trend of field distortion, e.g., 9.1% according to heterocharge accumulation in the vicinity of the anode. Incorporating α-NA and β-NA into the iPP matrix could provide improvement in field distortion but the mechanisms are different. For α-iPP samples, although the internal field just distorts by only 1.8%, DC conduction current and DC BDS become worse, largely due to the increase of DC conductivity and instable material performance. Therefore, higher conductivity benefits the charge dissipation process and low field distortion. For β-iPP samples, the maximum field distortion was successfully restricted by 2.8%, which should attribute to the newly-generated deep traps that greatly weakened the mobility of charge carrier in iPP materials [27]. It exhibits tremendous potential for the long-term running of high voltage DC power cable insulation.

**Figure 11:**
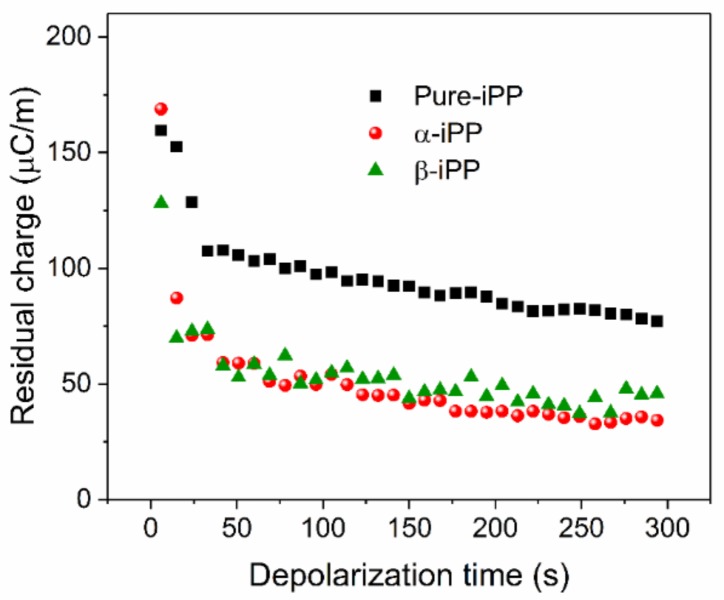
Residual charge in iPP samples after the removal of −100 kV/mm at room temperature.

To further demonstrate the ability of β-crystal to suppress space charge, the amount of residual charge was calculated according to the space charge distribution after the removal of −100 kV/mm at room temperature, shown in Figure 11. The residual charge was determined as follows [42]:

(4) $$Q(t)=\frac{1}{d}\int_{0}^{d}\left|\rho (x,t)\right|Sdx,$$

wherein *Q*(*t*) is the absolute value of charge amount per unit thickness at time *t*. *d* is the thickness. *ρ*(*x*,*t*) is the volume charge density at location *x* and time *t*. *S* is the area of the upper electrode. 

All curves exhibit a similar trend of residual charge in iPP samples, which could be divided into two parts with the depolarization time. The first part is located in the first ~30 s after depolarization, when injected charge is quickly detrapped, recombined, or diminished in the electrodes, which was regarded to exist in the shallow trapping sites [43]. Then, the dissipation rate of residual charge becomes slow and stable after ~30 s depolarization. At this stage, the amount of residual charge in Pure-iPP is the largest, leading to the larger internal field distortion.

## 4. Conclusions

A remarkable enhancement of electrical and impact strength is achieved by incorporating 0.2 wt % highly efficient TMB-5 β-NA into the iPP matrix. According to the morphology, thermal, mechanical, and electrical characterizations, β-NA mainly performs two functions. One is that the well-dispersed β-NA can act as a large density of nucleating sites, and subsequently induce a high relative content of β-crystal with more perfect structure, resulting in good toughness (25.3% higher than the unfilled counterparts) and elongation at break (200%), though the partial arrangement of β-crystal lead to a little loss of tensile strength. The other is that the interfacial regions among the β-crystal accompanying with a large decrease of the average size of crystalline agglomerates, thus resulting in the increase of charge trapping sites, thus enhancing DC breakdown strength, lowering space charge accumulation, and restricting internal charge distortion under a high external DC field of −100 kV/mm. This study provides a comprehensive explanation for the advantageous modification of β-NA over α-NA on the electrical property via melt mixing in iPP materials.

## Figures and Tables

**Table 1:** Crystalline parameters of iPP samples based on XRD and DSC results.

Sample	Crystalline Temperature (°C)	$\chi_{\alpha }$ (%)	$\chi_{\beta }$ (%)	$\chi_{\mathrm{all}}$ (%)	$\Phi_{\beta }$	$K_{\beta }$
Pure-iPP	118.1	42.6	0	42.6	0	0
α-iPP	127.5	43.1	0	43.1	0	0
β-iPP	124.6	12.0	30.3	42.3	0.72	0.81

**Table 2:** Shape and scale indexes of iPP samples under different voltage polarities.

Voltage Polarity	Sample	Shape Index	Scale Index (kV/mm)	Variation Percentage (%)
Negative DC	Pure-iPP	6.40	307.3	0
Negative DC	α-iPP	6.05	177.4	−42.3
Negative DC	β-iPP	10.49	340.1	+10.7
Positive DC	Pure-iPP	6.41	256.1	0
Positive DC	α-iPP	4.77	189.2	−26.1
Positive DC	β-iPP	5.72	331.0	+29.2
